# Effects of Depot Medroxyprogesterone Acetate Intramuscular Injection, Copper Intrauterine Device and Levonorgestrel Implant Contraception on Estradiol Levels: An Ancillary Study of the ECHO Randomized Trial

**DOI:** 10.3389/fgwh.2022.887541

**Published:** 2022-05-20

**Authors:** Rebecca Ryan, Aamirah Mussa, Mandisa Singtaa-Madliki, Joanne Batting, Yusentha Balakrishna, Chelsea Morroni, G. Justus Hofmeyr

**Affiliations:** ^1^Botswana Sexual and Reproductive Health Initiative, Botswana Harvard AIDS Institute Partnership, Gaborone, Botswana; ^2^Usher Institute, University of Edinburgh, Edinburgh, United Kingdom; ^3^Effective Care Research Unit, University of the Witwatersrand, Johannesburg, South Africa; ^4^Effective Care Research Unit, University of Fort Hare, Alice, South Africa; ^5^Biostatistics Unit, South African Medical Research Council, Durban, South Africa; ^6^MRC Centre for Reproductive Health, University of Edinburgh, Edinburgh, United Kingdom; ^7^Department of Obstetrics and Gynaecology, University of Botswana, Gaborone, Botswana; ^8^Eastern Cape Department of Health and Walter Sisulu University, Mthatha, South Africa

**Keywords:** contraception, randomized trial, depot medroxyprogesterone acetate, copper intrauterine device, levonorgestrel implant, estradiol

## Abstract

**Introduction:**

Hormonal contraception affects endogenous sex steroid levels. Robust evidence from randomized trials of the relative effects of different contraceptive methods is scarce. We compared the effects of three contraceptive methods on serum estradiol levels using data from women (18–35 years) requesting contraception in the Evidence for Contraceptive Options and HIV Outcomes (ECHO) randomized trial.

**Methods:**

Women were randomly allocated to the depot medroxyprogesterone acetate intramuscular (DMPA-IM) injection, copper intrauterine device (IUD) or levonorgestrel (LNG) implant. In this sub-study, stored baseline and 6-month serum samples were analyzed in 401 participants from East London, South Africa (DMPA-IM: 131, IUD: 135 and LNG: 135).

**Results:**

Baseline median (interquartile range, IQR) estradiol levels were similar between the three groups [DMPA-IM 229 (152–455), IUD 235 (168–426) and LNG 216 (153–419 pmol/L)]. At 6-months, median estradiol in the IUD group was unchanged (298 (163–467) pmol/L), whilst levels in the DMPA-IM and implant groups were significantly reduced from baseline. The median estradiol level in the DMPA-IM group [139 (97–193) pmol/L] was significantly lower than in both IUD (*p* < 0.0001) and implant (*p* = 0.005) groups; and level in the implant group [156 (112–250) pmol/L] was significantly lower than in the IUD group (*p* = 0.004).

**Conclusions:**

At 6-months (DMPA-IM nadir), median estradiol with DMPA-IM was 53% lower and with the LNG implant, 48% lower than with the IUD. The greater reduction in estradiol levels with the DMPA-IM injection compared to the LNG implant and IUD has implications for the relative psychological, sexual as well as physiological side-effects of these contraceptive methods.

**ECHO Study Registration:**

ClinicalTrials.gov, identifier: NCT02550067.

## Introduction

Hormonal contraception has complex effects on users' endocrine systems. Apart from the direct effects of the exogenous contraceptive hormones, circulating levels of endogenous sex steroid hormones are modified. It is important to have accurate data on the effects of alternative contraceptive methods in order to counsel women appropriately on the relative benefits and risks of different methods, to understand the potential clinical impact of these effects, and to guide future development of safer contraceptive methods.

Estrogens have multiple physiological effects such as on metabolism, menstruation, coagulation, bone health, the cardiovascular system (1) and the vaginal microbiome (2), and are also associated with adverse effects such as increasing susceptibility to candida albicans infection (3, 4). In addition, sex steroid hormones have important neuropsychological and behavioral effects. Hormones of the hypothalamic-pituitary-ovarian (HPO) axis are thought to play a role in depression (5). However, the relationships between sex hormones and psychological wellbeing are complex and poorly understood (6). Estradiol has neuromodulator effects on brain regions involved in mood and behavior (7). In a comprehensive 2021 systematic review, estradiol levels were found to be lower in women with premenstrual dysphoric disorder and postpartum depression, but not perimenopausal depression nor depression unrelated to reproductive transition phases (5).

Sexual function has been found to deteriorate with decreasing ovarian function, and to be improved by hormone replacement therapy with estrogen (8). Estradiol is also considered to play a positive role in sexual desire and arousal in premenopausal women (9). Use of hormonal contraception is associated with reduced libido and changes in sexual function. In a previous randomized trial, we found reduced sexual activity among participants randomized to injectable progestogens vs. the copper T 380 intrauterine device (IUD) (10, 11). The etonogestrel implant and the DMPA-IM have been associated with impaired sexual function ascribed to suppressed estrogen and/or androgen levels (12, 13).

Several observational studies have reported on estrogen levels in women choosing to use various hormonal contraception. Such studies are intrinsically subject to confounding. We are not aware of any studies comparing effects of the depot medroxyprogesterone acetate intramuscular (DMPA-IM) injection, the copper IUD and the levonorgestrel (LNG) implant on estradiol levels conducted in the context of a rigorous randomized clinical trial (RCT). The Evidence for Contraceptive Options and HIV Outcomes **(**ECHO)^1^ study (14, 15) presents a unique opportunity to compare hormonal levels between participants randomized to these methods.

## Materials and Methods

This is an ancillary study of the ECHO study, limited to participants enrolled at the Effective Care Research Unit site in East London, South Africa. The ECHO study protocol (15) and primary paper (14) have been published elsewhere. Briefly, Human immunodeficiency virus (HIV)-uninfected women requesting contraception aged 18–35 years who indicated that they had not used injectable hormonal contraception in the preceding 6 months, were randomly allocated to receive DMPA-IM, the copper IUD or the LNG implant, and were followed after 1 month then 3-monthly for 12 to 18 months. Blood samples were collected at baseline and at the 3-monthly visits, separated on site and the serum stored at −80°C in the BARC-SA Bio Repository in Johannesburg. For this ancillary study, only baseline and samples closest to 6 months were studied.

The original ECHO Trial was approved by ethics committees at all the participating sites. The application for additional tests on stored biological samples was approved by the Human Research Ethics Committee, University of the Witwatersrand on 6 May 2019, reference 141112. All women provided informed, written consent to authorize study participation.

### Laboratory Analysis

Laboratory tests were conducted by BARC-SA (Pty) Ltd. Estradiol (E2) levels were measured on stored samples for participants at baseline and closest available sample to 6 months, using a delayed one-step immunoassay for the quantitative determination of estradiol in human serum and plasma using chemiluminescent microparticle immunoassay (CMIA) technology. The few results above or below the detection level of the assay were assigned the value of the upper or lower limit of the assay respectively.

### Data Analysis

The laboratory data were entered into a secure Microsoft Excel spreadsheet, cleaned and merged with data from the original ECHO study database for analysis, using EpiInfo software. Baseline data were compared to ensure that the comparability of the groups was not compromised by loss to follow-up. Six-month values were compared with baseline values for each group. Pairwise comparisons were made between the three groups by allocated method at 6 months (intention to treat analysis). Statistical comparisons were by analysis of variance if the distribution of the data was normal, otherwise by the Mann-Whitney *U* test.

## Results

**Figure 1:**
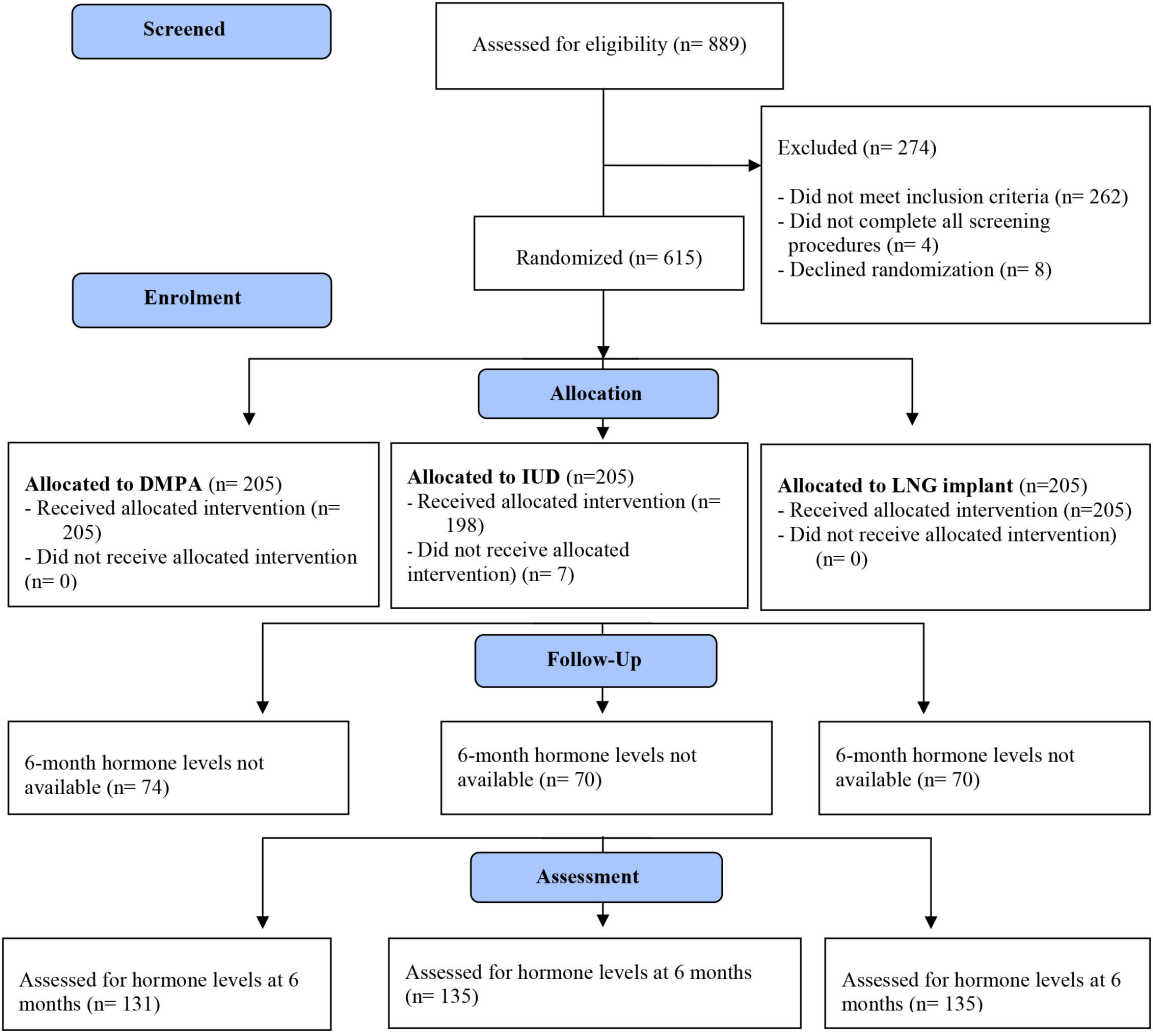
CONSORT flow diagram.

Of 615 participants enrolled at the East London site, results for hormonal studies were available for 401 (65%) participants both at baseline and at 6 months. The CONSORT flow diagram is shown in Figure 1. Baseline characteristics of participants are displayed in Table 1.

**Table 1:** Baseline variables.

	**DMPA-IM (*****n*** **=** **131)**		**Copper IUD (*****n*** **=** **135)**		**LNG implant (*****n*** **=** **135)**	
	**Mean**	**SD**	**Mean**	**SD**	**Mean**	**SD**
Age (years)	25.0	4.3	25.4	4.6	24.5	4.7
	***n***	**%**	***n***	**%**	***n***	**%**
Never married	122	93.1	131	97.0	131	97.0
Secondary school incomplete	75	57.3	63	46.7	75	55.6
Earns income	16	12.2	22	16.3	19	14.1
Nulliparous	33	25.2	40	29.6	41	30.4
Regular menses	76	58.0	89	65.0	79	58.5

*DMPA-IM, depot medroxyprogesterone acetate intramuscular injection; IUD, intrauterine device; LNG, levonorgestrel; SD, standard deviation*.

Retrospective random testing of baseline blood of 157/615 enrolled participants for evidence of recent hormonal contraceptive exposure found quantifiable medroxyprogesterone acetate in 85 (54%), levonorgestrel in 9 (5.7%), norethisterone in 7 (4.5%) and etonogestrel in 1 (0.6%). In view of the stringent randomization process, these effects are likely to be similar between the randomly allocated groups. Given the potential for distortion of baseline hormone levels due to prior contraceptive exposure, we primarily compared absolute differences between randomized groups at 6 months rather than change from baseline. Prior exposure to progestogen contraception would result in under-estimation of the changes observed in the progestogen group, as well as apparent changes in the copper IUD group in the opposite direction of the progestogen effects.

Baseline median (interquartile range, IQR) estradiol levels were similar between the three groups [DMPA-IM 229 (152–455) pmol/L, IUD 235 (168–426) pmol/L and LNG implant 216 (153–419) pmol/L] (Table 2). At 6 months, median estradiol level in the IUD group was unchanged [298 (163–467) pmol/L]. Median estradiol levels in the DMPA-IM and implant groups were significantly reduced from baseline. The median estradiol level in the DMPA-IM group [139 (97–193) pmol/L] was significantly lower than in both the IUD (*p* < 0.0001) and implant (*p* = 0.005) groups; and the level in the implant group [156 (112–250) pmol/L] was significantly lower than in the IUD group (*p* = 0.004).

**Table 2:** Estradiol results expressed as median values (interquartile range).

	**DMPA-IM**			**Copper IUD**			**LNG implant**			**DMPA vs. IUD**	**DMPA vs. implant**	**Implant vs. IUD**
**E2 pmol/L**	***n***	**Median**	**IQR**	***n***	**Median**	**IQR**	***n***	**Median**	**IQR**	***p***	***p***	***p***
Baseline	131	229	152–455	135	235	168–426	134	216	153–419	0.89	0.80*	0.40*
6 months	131	139	97–193	135	298	163–467	132	156	112–250	<0.0001*	0.005	0.004
P (change from baseline)		<0.0001*			0.51			0.0002*				

E2, estradiol; DMPA, depot medroxyprogesterone acetate; IUD, intrauterine device; LNG, levonorgestrel; CI, confidence interval; IQR, interquartile range; ^*^ Mann Whitney U test.

## Discussion

This study quantifies for the first time using RCT methodology the differences in levels of endogenous estradiol in young women (18–35 years) randomly allocated to receive the DMPA-IM injection, the LNG implant or non-hormonal contraception (the copper IUD). At 6 months, the median estradiol levels in women using both progestogen contraceptives were in the postmenopausal range. The striking finding was the significantly greater reduction in estradiol in women allocated to DMPA-IM injection than to the LNG implant. While the data for the LNG implant would represent the steady-state LNG effect, the samples were collected at the time of a DMPA-IM injection, and thus represented the nadir DMPA-IM effect. The average effect of DMPA-IM over time would be expected to be an even greater reduction in estradiol levels.

Our findings are consistent with a possible association of estradiol and sexual behavior. The main ECHO Trial [Appendix Table S11 of main trial paper (14)] reported sexual activity was generally lower with the LNG implant than with the IUD, and lower with DMPA-IM than with the LNG implant (14). The lower self-reported sexual activity with DMPA-IM than with the LNG implant are consistent with an ancillary study at three of the ECHO sites which found prostate-specific antigen levels in cervical samples to be less frequent in women allocated to DMPA than to the LNG implant (16).

Although an inclusion criterion for enrolment in the ECHO study was no injectable contraception in the last 6 months, retrospective spot checks conducted during the main study indicated that a proportion of women had evidence of persistent DMPA or norethisterone levels. Considerable discrepancies between self-reported and biologically confirmed prior contraceptive exposure have been reported in other studies (17). In addition, use of oral contraception was permitted up to the day preceding enrolment. It is therefore likely that some women in all groups had some estradiol suppression at baseline. This assumption is consistent with the (non-significant) increase in estradiol levels in the IUD group at 6 months. Therefore, the reductions from baseline estradiol in the hormonal method groups may be an under-estimate of the true suppressive effect of the methods. For this reason, while we have included the baseline estradiol levels to confirm comparability of the groups, we have primarily compared absolute levels at 6 months between groups rather than changes from baseline.

In conclusion, the significant reduction in endogenous estradiol with the LNG implant, and the significantly greater reduction with DMPA-IM have relevance to the estradiol-related physiological and psychological side-effects and beneficial effects of these methods. While data on the relationship of sex steroids to clinical parameters are not consistent, these effects might include menstrual cycle disruption, bone health, cardiovascular health, vaginal microbiome, candidiasis susceptibility, mood and sexual experience.

## Data Availability Statement

The data that support the findings of this ancillary study of the ECHO Study are available from the corresponding author upon reasonable request. Access will be granted if the concept is evaluated to have scientific merit and if sufficient data protections are in place. As of the time of publication, data access applications are in process with the governing institutional review boards of the ECHO Study to make de-identified data from the primary ECHO dataset publicly available. Requests to access these datasets should be directed to justhof@gmail.com.

## Ethics Statement

The studies involving human participants were reviewed and approved by Witwatersrand Human Research Ethics Committee and the WHO Research Ethics Review Committee. The patients/participants provided their written informed consent to participate in this study.

## Author Contributions

GH, MS-M, and JB were involved with conception, design, and conduct of the study. GH and YB performed the statistical analysis. GH wrote the first draft of the manuscript. RR, AM, and CM contributed to subsequent drafts. All authors have read and approved the manuscript.

## Funding

This work and the Evidence for Contraceptive Options and HIV Outcomes (ECHO) Study were made possible by the combined generous support of the Bill & Melinda Gates Foundation (Grant OPP1032115), the American people through the United States Agency for International Development (Grant AID-OAA-A-15–00045), the Swedish International Development Cooperation Agency (Grant 2017/762965–0), the South Africa Medical Research Council, and the United Nations Population Fund. Contraceptive supplies were donated by the Government of South Africa and US Agency for International Development. The hormonal assays were funded by a grant from Botswana Harvard Partnership.

## Author Disclaimer

The contents of this paper are solely the responsibility of the authors and do not necessarily reflect the views, decisions or policies of the institutions with which they are affiliated, the ECHO trial funders, or the supporting governments.

## Conflict of Interest

The authors declare that the research was conducted in the absence of any commercial or financial relationships that could be construed as a potential conflict of interest.

## Publisher's Note

All claims expressed in this article are solely those of the authors and do not necessarily represent those of their affiliated organizations, or those of the publisher, the editors and the reviewers. Any product that may be evaluated in this article, or claim that may be made by its manufacturer, is not guaranteed or endorsed by the publisher.

## Footnotes

^1^The Evidence for Contraceptive options and HIV Outcomes (ECHO) Trial was a multi-center, open label, randomized clinical trial comparing HIV incidence and contraceptive benefits in women using depot medroxyprogesterone acetate (DMPA-IM) injection, levonorgestrel (LNG) implants, and copper intrauterine devices (IUD). FHI 360 Study #523201.
